# Cognitive flexibility and DSM-5 severity criteria for eating disorders: assessing drive for thinness and duration of illness as alternative severity variables

**DOI:** 10.1186/s40337-023-00875-z

**Published:** 2023-09-11

**Authors:** Bernat Mora-Maltas, Ignacio Lucas, Roser Granero, Cristina Vintró-Alcaraz, Romina Miranda-Olivos, Isabel Baenas, Isabel Sánchez, Jessica Jiménez-del Toro, Jéssica Sánchez-González, Isabel Krug, Javier Tapia, Susana Jiménez-Murcia, Fernando Fernández-Aranda

**Affiliations:** 1 Clinical Psychology Unit, University Hospital Bellvitge and CIBERobn, Feixa Llarga s/n 08907 L’Hospitalet del Llobregat, Barcelona, Spain; 2 Psychoneurobiology of Eating and Addictive Behaviours Group, Bellvitge Biomedical Research Institute (IDIBELL), Barcelona, Spain; 3 CIBER de Fisiopatología de la Obesidad y Nutrición (CIBERobn), Instituto de Salud Carlos III, Barcelona, Spain; 4 Departament de Psicobiologia i Metodologia, Universitat Autònoma de Barcelona, Barcelona, Spain; 5 Melbourne School of Psychological Sciences, The University of Melbourne, Melbourne, Australia; 6 Gerencia Territorial Metropolitana Sud. Hospital Universitari de Bellvitge, Barcelona, Spain; 7 Department of Clinical Sciences, School of Medicine and Health Sciences, University of Barcelona, Barcelona, Spain

**Keywords:** Eating disorders, Neuropsychology, DSM-5-TR, Illness duration, Severity ratings

## Abstract

**Background:**

The severity criteria for eating disorders (EDs) proposed in the DSM-5 have been established without sufficient empirical support. Drive for thinness (DT) and duration of illness have been proposed as two alternative severity measures, however their empirical evidence is also limited. To date, no research has assessed the validity of current eating disorder (ED) severity criteria regarding cognitive flexibility factors. Cognitive flexibility is often impaired in EDs, becoming a possible severity symptom. The current study assessed for the first time (1) whether the severity indexes for EDs proposed in the DSM-5 were associated with deficits in cognitive flexibility and, (2) whether drive for thinness and illness duration, acted as an alternative, more meaningful severity indices for deficiencies in cognitive flexibility.

**Methods:**

Participants were 161 patients diagnosed with an ED, who were categorized according to DSM-5 severity categories, DT and duration of illness. Discriminative capacity of each classification was assessed for cognitive flexibility measured by Wisconsin card sorting test (WCST).

**Results:**

The findings for the DSM-5 classification comprised: (a) In the anorexia nervosa (AN) group, patients with moderate severity showed better scores in WCST than patients with mild and severe/extreme severity. Also, patients with moderate severity showed lower percentage of cognitive flexibility deficits than the other two severity categories; (b) For the binge spectrum disorders (BSD) group, the patients with mild severity showed a higher percentage of cognitive flexibility deficits than did the moderate and severe/extreme categories. When assessing the alternative severity index of DT, no differences were found in cognitive flexibility in any of the groups. Regarding illness duration, in the AN group the task performance of the patients with longer illness duration was worse than the performance of the short duration group and, in the BSD group, patients with longer duration also showed more deficits in cognitive flexibility than the patients with shorter duration of illness.

**Conclusions:**

Our findings point out the limitations of the DSM-5 severity criteria to categorize cognitive flexibility in EDs and support illness duration as an alternative severity approach for EDs.

## Abstract (highlights)

The current severity criteria for eating disorders were defined without enough evidence and present several limitations. Therefore, some researchers proposed alternative criteria like the drive for thinness or the duration of the disorder. Eating disorders are characterized by cognitive impairments such as deficits in cognitive flexibility. Cognitive flexibility is the ability to adapt our behaviour to the needs of the environment. According to our results, current severity criteria do not classify correctly in terms of cognitive flexibility. Moreover, this study shows that patients with longer illness duration show less cognitive flexibility. Therefore, this study highlights the limitations of current severity criteria for eating disorder to classify according to cognitive flexibility. Our findings also show the importance of taking into account the illness duration in order to assess the severity of the disorder.

## Background

The fifth edition of the Diagnostic and Statistical Manual of Mental Disorders (DSM-5) [1] and its text revised version (DSM-5-TR) [2] propose four different severity gradients for eating disorders (EDs) (‘mild’, ‘moderate’ ‘severe’ and ‘extreme’). The severity of the disorder is determined by the body mass index (BMI = kg/m^2^) for anorexia nervosa (AN). Regarding binge spectrum disorders (BSD), a classification previously used in the literature [3] that includes bulimia nervosa (BN) and binge eating disorder (BED), the severity is determined by the weekly frequency of episodes of inappropriate compensatory behaviours for BN, and the number of weekly binge episodes for BED. However, a recent systematic review and meta-analysis presented a compound of scientific evidence that acknowledged the limitations of the current severity classification for EDs [4]. According to the literature, the criteria and cut-offs used for defining the severity of EDs are controversial and lack sufficient empirical support [5–10].

With respect to AN, inconsistent results have been found, maybe because using only BMI does not take into account important factors such as physical (e.g. weight history), psychological or cognitive factors that may reflect different kinds of impairment [11–13]. In this line, several studies assessing individuals with AN did not find significant variation among the different DSM-5 severity groups regarding distress, psychiatric comorbidity or further attitudinal eating disorder (ED) symptoms (i.e., shape concerns/weight) [5, 6, 9, 14–18]. Moreover, Dakanalis et al. [19] found that patients with less severe AN showed more bingeing and purging behaviours than the ones with more severity, and the presence of binge-purge behaviours in AN have been associated with more psychopathology [20–22], more relapses [23] and poorer treatment outcomes [24]. For BSD, incongruous findings regarding the utility of the DSM-5 severity index have been obtained. Studies that support the proposed DSM-5 criteria found that patients with severe and extreme BN had more psychiatric comorbidities, functional impairments, perfectionism, and ED body-related attitudes and behaviours than those in the mild and moderate categories [17, 25–28]. Conversely, a study by Zayas et al. [18] supported the utility of the BN severity index with respect to ED psychopathology in females, but not in males, and other studies found rarely or no differences for these variants across the BN severity groups [5, 29, 30]. Furthermore, in some other studies few patients diagnosed with BN fell into the severe or extreme categories, raising doubts about the cut-off points of the BN classification [17, 31, 32]. The literature has also been contradictory with regard to BED. Some studies reported differences between severity groups in relation to BMI, ED characteristics, comorbidity (personality disorders, biases and emotional difficulties), and other factors of distress and impairment [5, 11, 27, 33, 34]. However, various studies found no differences between severity categories with respect to psychiatric comorbidity, prognostic prediction or body attitude [6, 8, 11, 17, 30, 34, 35]. In addition, as seen in BN, few individuals with BED fall into the severe or extreme BED categories [4–6, 8, 17].

Due to the uncertainty that exists regarding the functionality of the DSM-5 severity index, researchers have introduced transdiagnostic indices to find alternative severity classifications for each ED subtype [4]. In this line, previous research has proposed other alternative measures for ED severity to overcome the limitations presented by the DSM-5 classification. For example, Krug et al. [6] proposed an alternative transdiagnostic indicator of ED severity, the *drive for thinness* (DT) dimension of the eating disorders inventory-2 (EDI-2) [36]. DT is defined as an extreme fear of weight gain, which reinforces disordered eating patterns (especially restrictive eating) [37–39]. However, DT is just one factor involved in the complex process of EDs [40].

*Duration of illness* has also been reported as an alternative indicator of severity for EDs. Specifically, the EDs literature has repeatedly demonstrated that a shorter duration of illness is related to a more favourable outcome of treatment for EDs [41–44]. For instance, a study by Fernández-Aranda et al. [45] has shown that duration of illness was linked with poor response to treatment, suggesting that the duration of illness could be a good marker of severity. In this study, the duration cut-off points from which there would be a greater risk of having poor results were: 12 years for patients diagnosed with AN, 13 years for patients with BN, and 21 years for patients with BED. In addition, other empirical studies have reported an association between the duration of illness and deficits in cognitive flexibility in AN [46] and BSD [47].

Cognitive flexibility is defined as the ability to adjust individual's beliefs or behaviour in response to new situations, which is essential for behaviour self-regulation [48]. A common neuropsychological task to measure cognitive flexibility is the Wisconsin Card Sorting Test (WCST; [49]). Although the role of cognitive flexibility in EDs is still being studied, there is increasing support that this cognitive malfunction may be one of the factors that influences the development and maintenance of EDs. A meta-analysis carried out by Wu et al. [50] reported cognitive flexibility impairments in patients with restrictive subtype of AN, BN and BED. In addition, a recent study associated AN and BSD with impairments in different executive domains, including cognitive flexibility [51]. In that study, poor cognitive performance correlated with anxious, depressive, and ED symptoms. Other results also associated cognitive flexibility impairments in EDs with comorbid symptomatology, such as depression or anxiety [52]. In patients with AN, lack of cognitive flexibility has been considered a neurocognitive endophenotype that may contribute to compulsive and rigid behaviour [53–55]. Specific treatments, such as the cognitive remediation therapy, are aimed at the cognitive impairments shown by these patients [56–58]. Complement usual treatment with cognitive remediation therapy have proven to produce a significant improvement in eating disorder-specific health-related quality of life and a greater reduction of eating disorder psychopathology [59]. However, the efficacy of cognitive remediation therapy remains unknown due to a lack of conclusive data from other studies, which have found no evidence that this treatment improves eating disorder symptoms [60, 61]. For BSD, a meta-analysis reported that deficits in cognitive flexibility are associated with the inability to stop compulsive overeating [50]. Moreover, poor performance of BSD patients in the WCST have proven to be a predictor of bad treatment outcome [62]. However, in BSD there are inconsistent results, with studies reporting deficits in cognitive flexibility [50, 63], and others finding no significant differences [64, 65] compared to controls.

Until now, the literature has taken little account of cognitive factors as an effective measure to assess the severity of EDs. In fact, to our knowledge, there are no studies that evaluate cognitive deficits of patients with an ED based on the DSM-5 severity indices.

### The current study

The current study assessed, for the first time: (1) Whether the severity indices for EDs proposed in the DSM-5 were associated with deficits in cognitive flexibility and, (2) Whether DT and illness duration, acted as an alternative, more meaningful severity indices for deficiencies in cognitive flexibility.

## Methods

### Participants

The participants were 161 adults (130 females and 31 males) who met the DSM-5 criteria for an ED. All patients were diagnosed by experienced psychologists. Those who were diagnosed according to DSM-IV-TR criteria [66] were reanalysed and recoded post-hoc using DSM-5 criteria [2]. Most patients were treatment naïve (*n* = 116, 72%). The number of patients with one previous treatment was *n* = 24 (14.9%), with two previous treatments was *n* = 10 (6.2%), and with three or more previous treatments *n* = 11 (6.8%). There was a positive correlation between the number of previous treatments and illness duration (non-parametric correlation = 0.333) in the whole sample. The distribution of the ED diagnoses was: 72 AN-Restrictive (AN-R), 28 AN-Binge-eating/Purging type (AN-BP), 34 BN, and 27 BED. Patients with a diagnosis of AN-R and AN-BP were categorized in the AN group, and patients diagnosed with BN and BED were categorized in the BSD group. This classification was based on the common physiological and psychological factors that differentiate ED subtypes [3]. The AN group had a mean age of 27.28 years (SD = 8.99). The BSD group had a mean age of 35.62 years (SD = 10.64). The age range of the total sample is between 17 and 58 years. Table 1 displays a detailed sociodemographic description of each group, and the result of the statistical comparison. Exclusion criteria were having an intellectual disability, the presence of an organic mental disorder or an active psychotic disorder.

**Table 1:** Sample description

	AN group (*n* = 100)		BSD group (*n* = 61)		*p*
	*n*	%	*n*	%	
*Sex*					
Females	92	92.0	38	62.3	< 0.001*
Males	8	8.0	23	37.7	
*Marital status*					
Single	81	81.0	36	59.0	0.006*
Married	15	15.0	16	26.2	
Divorced	4	4.0	9	14.8	
*Education*					
Primary	33	33.0	26	42.6	0.324
Secondary	38	38.0	23	37.7	
University	29	29.0	12	19.7	
*Employment*					
Unemployed	43	43.0	22	36.1	0.384
Employed/student	57	57.0	39	63.9	
*Social position*					
High	12	12.0	4	6.6	0.028*
Mean-high	21	21.0	20	32.8	
Mean	24	24.0	23	37.7	
Mean-low	34	34.0	9	14.8	
Low	9	9.0	5	8.2	

	Mean	SD	Mean	SD	*p*
Age (years old)	27.28	8.99	35.62	10.64	< 0.001*
Onset ED (years old)	21.06	8.58	25.21	11.38	0.009*
Duration ED (years)	6.22	6.30	10.41	8.12	< 0.001*
EDI-2 drive for thinness	9.72	7.31	14.38	5.12	< 0.001*
Body mass index (kg/m^2^)	16.21	1.50	30.94	8.93	< 0.001*
Inappropriate compensatory behaviours	2.67	7.84	4.69	6.57	0.094

*AN* anorexia nervosa group (*AN-R* anorexia nervosa restrictive, *AN-BP* anorexia nervosa binge-purging), *BSD* binge spectrum disorders symptoms group (*BN* bulimia nervosa, *BED* binge eating disorder), *ED* eating disorder, *SD* standard deviation, *EDI-2* eating disorders inventory-2; Inappropriate compensatory behaviours: number of vomits, laxatives and diuretics per week. *Bold: significant comparison

Data were collected between November 2007 and January 2020 at the Eating Disorders Unit of the Bellvitge University Hospital (Barcelona, Spain). All participants received information about the procedure and signed an informed consent form. All procedures were approved by the Ethical Committee of the Bellvitge University Hospital in accordance with the Helsinki Declaration of 1975 as revised in 1983 (Refs. 34/05, 307/06).

### Sociodemographic and clinical information

Sociodemographic data were collected from each participant. These data included age, education level, marital status and employment, as well as ED onset and duration. Sex data is also reported, showing a higher proportion of females, but in accordance with prevalence estimates of EDs [67]. Social position was calculated using the Hollinghead method [68].

### Psychological assessment

Participants were evaluated using the DT subscale of the Eating Disorders Inventory-2 (EDI-2) [36]. This questionnaire evaluates cognitive and behavioural features related to the ED. DT factor is defined as the extreme fear of weight gain and over-preoccupation about diet and weight. In this study, Cronbach’s alpha for the EDI-2 DT subscale was 0.884 (indicating good internal consistency).

### Neuropsychological assessment

The computerized version of the Wisconsin Card Sorting Test (WCST) [49, 69] was used to evaluate cognitive flexibility. The WCST includes 128 cards that comprise three available categories: number (*N*), colour (*C*) and shape (*S*). For a right pair, participants must identify the sorting rule, receiving the feedback of “Right” or “Wrong” after each sort. By trial and error, the participant must learn to change the sorting categories according to the given feedback. Initially, *C* is the correct sorting category, and positive feedback is given only if the card is placed in the pile with the same colour. After 10 consecutive right pairs, the rule is changed, and then, another sorting rule must be identified. There are up to six attempts to detect the sorting rule and five rule shifts during the task. Each rule attainment is referred to as “category completed”. The task ends when all 128 cards are sorted or after the six full categories are completed. The number of completed categories is recorded, as well as the percentages of errors, perseverative responses, perseverative errors, non-perseverative errors, and conceptual level responses. The presence of deficits in cognitive flexibility was determined by scores below the 16^th^ percentile in any of the WCST scales perseverative errors, non-perseverative errors, and number of completed categories, based on normative data published in the manual of the task and in accordance with previous literature about cognitive functions [70, 71]. The method to calculate presence of deficits in cognitive flexibility is not described in the manual, but have been previously used in eating disorders [47].

### Severity index categorization

#### DSM-5

The DSM-5 severity classifications were carried out following the criteria proposed in the manual [1]. For the AN group, four severity categories were defined according to patients’ BMI: mild (> 17.0 kg/m^2^), moderate (16–16.99 kg/m^2^), severe (15–15.99 kg/m^2^) o extreme (< 15 kg/m^2^). The severity of the ED in patients diagnosed with BN was defined by the number of inappropriate compensatory behaviours per week (vomits, laxatives and diuretics): mild (1–3 episodes/week), moderate (4–7 episodes/week), severe (8–13 episodes/week) and extreme (> 14 episodes/week). In patients with a BED diagnosis, the severity categories were defined by the same categorization as for BN but taking into account binge eating episodes per week instead of compensatory behaviours.

#### Drive for thinness

Krug et al. [6] used an alternative categorization for ED severity based on DT symptomatology using the EDI-2 DT subscale. We used the same cut-off point for classifying low DT (≤ 14) participants and high DT (> 14) participants, based on the recommendations by [72] for screening purposes. This cut-off point has also been used in other previous studies [73].

#### Illness duration

Illness duration cut-off points were based on a previous recent study that highlighted the importance of the duration of the disorder in the treatment outcome of EDs [45]. This previous study calculated the duration cut-off points from when there would be a higher risk of having poor treatment outcomes in each subtype of ED. Hence, the cut-off points used in this study were 12 years for patients diagnosed with AN-R and AN-BP, 13 years for patients with BN, and 21 years for patients with BED.

### Procedure

Evaluations were conducted in two separate sessions prior to the psychological treatment. In the first one, we collected sociodemographic data and conducted the psychological assessment. And, in the second one, participants completed a computerized version of the WCST [49, 69].

### Statistical analysis

The statistical analysis was performed with Stata17 (Stata Press, 2021) for Windows. Chi-squared tests (χ^2^) were done for the comparison of categorical variables between the groups (e.g. cognitive flexibility deficits), and analysis of variance (ANOVA) was done for the comparison of quantitative measures (e.g. neuro-psychological scores). The effect size of the proportion and mean comparisons was estimated through Cramer's-V coefficient for categorical variables [the thresholds 0.06, 0.15 and 0.30 were considered for low/poor effect size, moderate/medium, and high/large (Cohen, 1998)] and partial eta-squared coefficient (η2) for quantitative measures [the thresholds for low/poor, moderate/medium and high/large were 0.06, 0.10, and 0.25 (Levine and Hullett, 2002)]. Increase of Type-I error due to multiple comparisons was controlled using the Finner Method (Finner, 1993), a family-wise procedure that has proved to be more powerful than the classical Bonferroni correction.

## Results

### Severity distribution

**Fig. 1:**
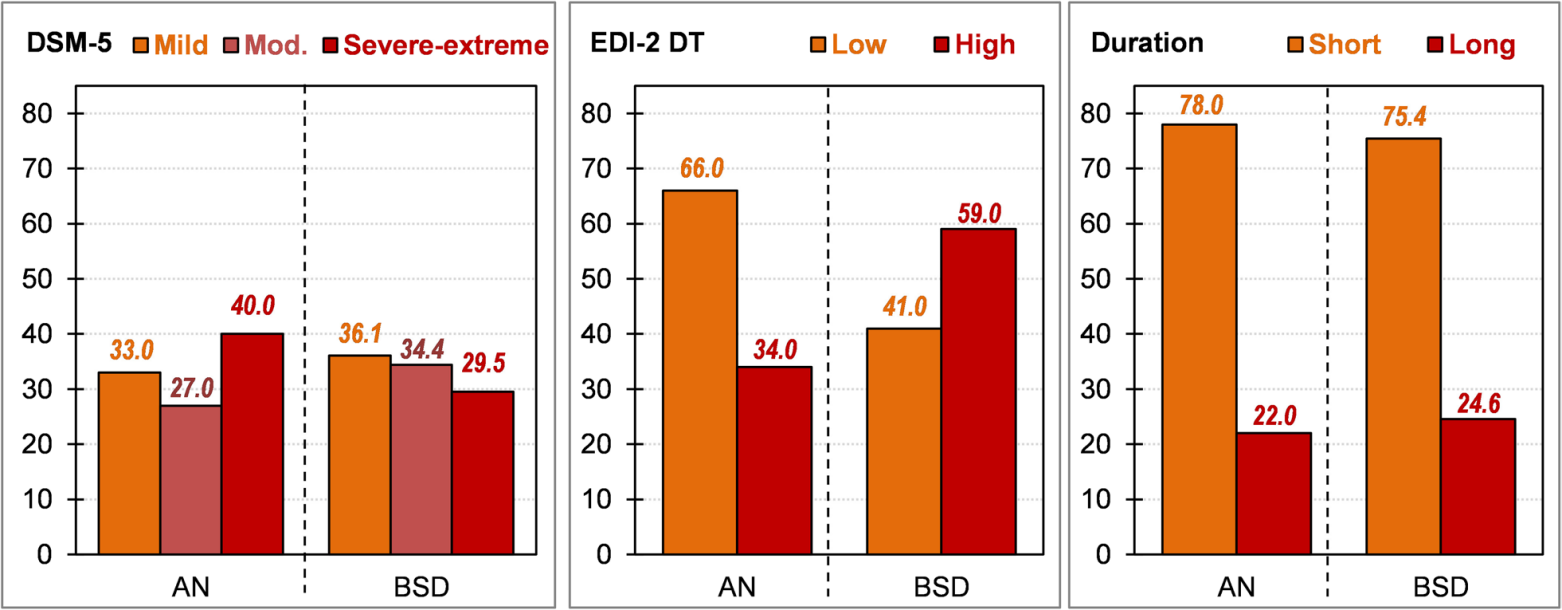
Severity prevalence estimates according to the three classification methods of the study (DSM-5, drive for thinness and illness duration)

Figure 1 displays bar-charts for the severity levels according to the three classification methods of the study (DSM-5, DT and illness duration). Among patients in the AN group, the more prevalent categories were: severe-extreme severity based on the DSM-5 severity levels (grouping 40.0% of patients), low score based on the EDI-2 DT scale (66.0%), and short duration (78.0%) based on the illness duration. Among patients of the BSD group, the more prevalent categories were mild severity for the DSM-5 classification system (36.1%), high score based on the EDI-2 DT scale (59.0%), and short duration based on the illness duration (75.4%).

### Cognitive flexibility based on the DSM-5 severity classification

Table 2 shows the discriminative capacity of the classification methods for cognitive flexibility based on DSM-5 severity criteria. Among patients in the AN group, the moderate severity group classified by the DSM-5 criteria showed better performance in WCST (PctErrors, PctPersRsps, PctPersErrors, PctCLRsps) than the mild and severe/extreme groups. This classification also reported that patients grouped in the mild and severe/extreme categories showed a higher percentage of deficits in cognitive flexibility than the moderate group. Among BSD group, patients classified in the mild severity category showed higher percentage of deficits in cognitive flexibility than the moderate and severe/extreme categories.

**Table 2:** Discriminative capacity on cognitive flexibility for the DSM-5 severity classification

AN group (n = 100)											
*WCST T-scores*	*DSM-5 criteria*										
	Mild (G1) *n* = 33		Mod(G2) *n* = 27		S/E (G3) *n* = 40		Factor group		Pairwise comparisons		
									*G*1–*G*2	*G*1–*G*3	*G*2–*G*3
	*Mean*	*SD*	*Mean*	*SD*	*Mean*	*SD*	*p*	ES	*p*	*p*	*p*
WCST: PctErrors	45.36	11.37	52.44	10.31	46.30	11.24	0.033*	0.068	0.015*	0.719	0.028*
WCST: PctPersRsps	46.91	11.91	55.11	10.71	46.33	10.59	0.004*	**0.108**^**†**^	0.005*	0.823	0.002*
WCST: PctPersErrors	46.91	11.91	53.74	10.45	46.10	10.63	0.016*	0.082	0.019*	0.756	0.006*
WCST: PctNonPersErrors	45.67	10.69	51.70	10.41	47.23	9.86	0.072	0.053	0.026*	0.521	0.084
WCST: PctCLRsps	44.79	11.75	52.37	10.15	46.05	11.62	0.026*	0.072	0.011*	0.636	0.027*
*Deficit*	*n*	*%*	*n*	*%*	*n*	*%*	*p*	ES	*G*1*–G*2	*G*1*–G*3	*G*2–*G*3
Cognitive flexibility	13	40.6%	2	7.4%	11	27.5%	0.015*	**0.291**^**†**^	0.004*	0.240	0.041*

BSD group (n = 61)											
*WCST T-scores*	*DSM-5 criteria*										
	Mild (G1) *n* = *22*		Mod(G2) *n* = *21*		S/E (G3) *n* = *18*		Factor group		Pairwise comparisons		
									*G*1–*G*2	*G*1–*G*3	*G*2–*G*3
	*Mean*	*SD*	*Mean*	*SD*	*Mean*	*SD*	*p*	ES	*p*	*p*	*p*
WCST: PctErrors	41.68	13.12	46.05	9.62	47.94	11.22	0.209	0.052	0.216	0.091	0.608
WCST: PctPersRsps	44.18	14.71	47.05	11.13	50.06	16.48	0.431	0.029	0.510	0.197	0.511
WCST: PctPersErrors	42.95	14.60	46.81	10.98	49.28	16.76	0.367	0.034	0.377	0.166	0.590
WCST: PctNonPersErrors	43.00	11.56	46.86	7.95	48.78	9.06	0.163	0.061	0.198	0.066	0.540
WCST: PctCLRsps	41.73	12.71	45.81	9.71	47.89	11.27	0.219	0.051	0.242	0.092	0.570
*Deficit*	*n*	*%*	*n*	*%*	*n*	*%*	*p*	ES	*G*1–*G*2	*G*1–*G*3	*G*2–*G*3
Cognitive flexibility	11	50.0%	5	23.8%	2	11.1%	0.021*	**0.355**^**†**^	0.046*	0.009*	0.303

*AN group* anorexia nervosa group, *BSD* group binge spectrum disorders group, *WCST* Wisconsin card sorting test, *PctErrors* percentage of errors, *PctPersRsps* percentage of perseverative responses, *PctPersErrors* percentage of perseverative errors, *PctNonPersErrors* percentage of non-perseverative errors, *PctCLRsps* percentage of conceptual level responses, *Mod* moderate, *S/E* severe/extreme, *SD* standard deviation, *ES* effect size [partial eta-squared for ANOVA (η^2^) and Cramer-V for chi-square test (C-V)]; *Bold: significant comparison; ^†^Bold: effect size into the mild/moderate to the high/large range (η^2^ > 0.10 or C-V > 0.15).

### Cognitive flexibility based on alternative severity classifications

Table 3 shows the discriminative capacity of alternative severity classification methods for cognitive flexibility. No differences in cognitive flexibility were obtained for the DT classification in any group. However, regarding duration of the disorder, patients of the AN group and a short duration of the disorder showed a better performance in WCST (PctErrors, PctPersRsps, PctPersErrors, PctNonPersError, PctCLRsps) than the patients with a longer duration. In the patients of the BSD group classified by duration of illness, no differences were found in the task scores. Nevertheless, the duration of the illness differentiated the presence of deficits in cognitive flexibility in patients with BSD, with a higher percentage of deficits in cognitive flexibility for the patients with longer duration.

**Table 3:** Discriminative capacity on cognitive flexibility for alternative severity classifications

AN group (n = 100)												
*WCST T-scores*	EDI-2 drive for thinness						Duration of the disorder					
	Low *n* = 66		High *n* = 34		Factor group		Short *n* = 78		Long *n* = 22		Factor group	
	*Mean*	*SD*	*Mean*	*SD*	*p*	ES	*Mean*	*SD*	*Mean*	*SD*	*p*	ES
WCST: PctErrors	46.94	11.36	49.03	11.29	0.385	0.008	49.13	10.92	42.41	11.44	0.013*	0.061
WCST: PctPersRsps	48.62	11.43	49.41	12.09	0.749	0.001	50.54	11.44	43.05	10.43	0.007*	0.072
WCST: PctPersErrors	48.08	11.25	49.12	11.79	0.667	0.002	50.01	11.15	42.82	10.64	0.008*	0.069
WCST: PctNonPersError	47.14	10.45	49.44	10.47	0.299	0.011	49.01	10.38	44.05	10.06	0.049*	0.039
WCST: PctCLRsps	46.59	11.65	48.79	11.54	0.371	0.008	48.78	11.21	42.23	11.76	0.018*	0.055
*Deficit*	*n*	*%*	*n*	*%*	*p*	ES	*n*	*%*	*n*	*%*	*p*	ES
Cognitive flexibility	19	29.2%	7	20.6%	0.353	0.093	18	23.4%	8	36.4%	0.222	0.123

BSD group (n = 61)												
*WCST T-scores*	EDI-2 drive for thinness						Duration of the disorder					
	Low *n* = 25		High *n* = 36		Factor group		Short *n* = 46		Long *n* = 15		Factor group	
	*Mean*	*SD*	*Mean*	*SD*	*p*	ES	*Mean*	*SD*	*Mean*	*SD*	*p*	ES
WCST: PctErrors	46.32	11.98	44.14	11.36	0.474	0.009	46.43	11.74	40.73	10.21	0.098	0.046
WCST: PctPersRsps	48.40	13.11	45.86	14.89	0.495	0.008	48.80	14.95	41.07	9.42	0.065	0.057
WCST: PctPersErrors	47.68	12.99	45.08	15.05	0.487	0.008	48.13	14.99	40.07	9.33	0.055	0
WCST: PctNonPersErrors	46.40	10.16	45.78	9.75	0.810	0.001	47.09	9.97	42.80	9.01	0.144	0.036
WCST: PctCLRsps	46.68	11.90	43.75	11.10	0.329	0.016	46.35	11.57	40.67	10.16	0.095	0.047
*Deficit*	*n*	*%*	*n*	*%*	*p*	ES	*n*	*%*	*n*	*%*	*p*	ES
Cognitive flexibility	9	36.0%	9	25.0%	0.354	0.119	10	21.7%	8	53.3%	0.020*	**0.298**^**†**^

*AN group* anorexia nervosa group, *BSD group* binge spectrum disorders group, *WCST* Wisconsin card sorting test, *PctErrors* percentage of errors, *PctPersRsps* percentage of perseverative responses, *PctPersErrors* percentage of perseverative errors, *PctNonPersErrors* percentage of non-perseverative errors, *PctCLRsps* percentage of conceptual level responses, *Mod* moderate, *S/E* severe/extreme, *SD* standard deviation, *ES* effect size [partial eta-squared for ANOVA (η^2^) and Cramer-V for chi-square test (C-V)]; *Bold: significant comparison; ^†^Bold: effect size into the mild/moderate to the high/large range (η^2^ > 0.10 or C-V > 0.15).

## Discussion

A first aim of the present study was to determine if the DSM-5 severity criteria for EDs were able to assess the presence of deficits in cognitive flexibility, considering that it could be a core symptom of severity in EDs. Our second aim was to evaluate whether other alternatives variables such as DT or illness duration could be associated with poorer cognitive flexibility. Using the DSM-5 severity criteria for AN, we observed that the moderate severity group performed better in the WCST than the mild and severe/extreme groups, which presented a similar performance. In the same line, we found that patients classified in the mild and severe/extreme groups according to DSM-5 criteria, presented a higher percentage of deficits in cognitive flexibility than the moderate severity group. Considering the BSD group, our study did not find significant differences in cognitive flexibility performance between the DSM-5 severity groups. Additionally, the less severe group showed more cognitive flexibility deficits than the other two groups. These results suggest that DSM-5 severity criteria were not able to discriminate between cognitive flexibility levels of patients diagnosed with a BSD. Regarding both clinical groups, the present findings showed that DT did not discriminate poor cognitive flexibility. However, duration of illness did present discriminative capacity to assess poor cognitive flexibility, resulting in an alternative severity classification for EDs. In the AN group, the WCST performance of the long duration group was worse than the performance of the short duration group. Similarly, in the BSD group, the long duration group included a higher percentage of people with cognitive flexibility deficits.

These results illustrate that the DSM-5 severity ratings for AN, that are exclusively based on BMI, do not correspond to the deficits in cognitive flexibility. In patients diagnosed with AN, poor cognitive flexibility may be associated with the perseveration of maladaptive cognitive and behavioural patterns [74–76]. Therefore, it may contribute to the maintenance of the fixation on weight loss, weight control and calorie counting, excessive exercise routines, or instilled body image distortion [77, 78], all of which are clinical symptoms in AN [30, 79]. Our findings are consistent with previous research that highlighted the limited clinical utility of DSM-5 severity specifiers for AN [4, 5, 9, 18]. Considering the BSD group, taking into consideration that poor cognitive flexibility is frequently linked to the inability to cut off compulsive overeating [50] and difficulties set shifting attention away from ED-related stimuli [80], these findings are consistent with other studies that highlight the limited clinical support for DSM-5 severity criteria for BN [5, 30, 81] and BED [8, 11, 34, 35]. The results highlight the limitations of the DSM-5 severity criteria for EDs, as there are important domains, such as cognitive flexibility, that do not map onto the current, linear severity criteria. In this particular sample, cognitive flexibility seems to be associated with other factors, such as the duration of illness.

The present results suggest that duration of illness could be a better variable than DSM-5 severity criteria to identify poor cognitive flexibility in patients diagnosed with an ED. The poor cognitive flexibility presented by the two clinical groups with longer duration of illness could be suggested as a common EDs feature. Some studies have associated difficulties in cognitive flexibility with a fixed idea and rigid eating style based on idiosyncratic rules and with a greater resistance to be modified by therapy [78, 82]. Therefore, it seems that in EDs, the difficulty to adapt to new behaviour and rules in a changing environment could be associated with longer duration of the disorder. In addition, cognitive rigidity is a variable that is at odds with the notion of change and is likely to present difficulties to therapy and, therefore, is considered one of the factors associated with a worse prognosis [74]. In fact, in EDs, the lack of response to treatment has been linked to the illness duration and therefore, with chronicity [45]. Regarding duration and cognition, while some studies have observed that longer illness duration and severity in ED symptomatology were associated with executive dysfunctions [47, 83, 84], other studies did not find significant associations between longer illness duration and cognitive deficits [85, 86]. Therefore, the literature on the impact of illness duration on executive functions requires further investigation.

The preceding leads us to suggest that the DSM severity classification may not adequately reflect the severity from a neurocognitive point of view. Consequently, it may be beneficial to consider alternative factors in order to define severity classifications for EDs. Our results do not support DT as a transdiagnostic measure of severity for EDs. This variable may present some limitations as a transdiagnostic measure to assess eating disorders severity as, for example, some individuals could have different concerns, such as muscularity [87]. According to our results, duration of illness seems to be a better severity variable in terms of cognitive deficits. Although cognitive features are not yet considered an important severity measure in EDs, several studies have focused intervention on improving executive functions as alternative treatment with promising results [57, 88]. Therefore, the improvement of executive functions, such as cognitive flexibility, could influence a greater ability to adapt and guide problematic behaviours. However, a wide range of commonly shared features (i.e. affective, cognitive, biological, or personality) that can occur across all ED diagnoses have to be taken into account as well.

### Limitations and strengths

The results of this study must be interpreted in light of its limitations. First, our sample size was limited to test the discriminative power of DSM-5 severity criteria regarding cognitive flexibility across all ED subtypes. Second, related to the previous one, due to the heterogeneity of each group it is not possible to reach conclusions about differences between all the ED subtypes included in the study (e.g., BN, BED). Future studies should include a sufficient sample of each subtype to better identify the characteristics of each. Third, people diagnosed with an ED usually present deficits in different cognitive domains, hence, other cognitive domains could also have been explored, such as decision making or working memory. Fourth, although the study used for establishing the duration thresholds is representative of the population included in this study, using the thresholds proposed in a single study could represent a limitation. Fifth, it is important to highlight that this study included only a treatment seeking adult ED population. Consequently, our findings might not generalize to other populations, such as adolescents with an ED, for which some studies [89] revealed that cognitive impairment was not linked to AN. Future studies need to verify the DSM-5 severity index for EDs and other transdiagnostic severity indicators across a range of different treatment seeking and community samples.

However, our study also presents some remarkable strengths. First, our results are in line with previous studies that reported the limitations of the DSM-5 severity criteria. Moreover, this study includes further evidence that strengthens the conclusions derived from those studies, because these limitations seem to extrapolate to cognitive domains such as cognitive flexibility. Second, these results encourage the application of a transdiagnostic severity indicator based on illness duration.

## Conclusions

The present study makes noteworthy contributions to evidence the limitations of the DSM-5 and DSM-5-TR severity criteria for EDs. The proposed severity classification does not demonstrate good discrimination in terms of cognitive flexibility levels, a core significant feature of EDs. Furthermore, our findings show that the ED duration is associated with cognitive flexibility deficits, confirming that illness duration can be a good marker of severity in EDs. Future studies should aim to further demonstrate all the limitations of the DSM-5 and DSM-5-TR severity classification for EDs and also to propose alternative severity variables.

## Data Availability

The datasets generated during and/or analyzed during the current study are not publicly available due to ethical restrictions in order to protect the confidentiality of the participants, but are available from the corresponding author on reasonable request.

## Abbreviations

AN: Anorexia nervosa
ANOVA: Analysis of variance
AN-BP: Anorexia nervosa, binge-purgative subtype
AN-R: Anorexia nervosa, restrictive subtype
BED: Binge eating disorder
BMI: Body mass index
BN: Bulimia nervosa
BSD: Binge spectrum disorders
DT: Drive for thinness dimension of the eating disorders inventory-2
DSM-5: Diagnostic and statistical manual of mental disorders, fifth edition
DSM-5-TR: Diagnostic and statistical manual of mental disorders, fifth edition, text revision
ED: Eating disorder
EDs: Eating disorders
EDI-2: Eating disorders inventory-2
ES: Effect size
Mod: Moderate severity
PctErrors: Percentage of errors in the Wisconsin Card Sorting Test
PctPersRsps: Percentage of perseverative responses in the Wisconsin Card Sorting Test
PctPersErrors: Percentage of perseverative errors in the Wisconsin Card Sorting Test
PctNonPersErrors: Percentage of non-perseverative errors in the Wisconsin Card Sorting Test
PctCLRsps: Percentage of conceptual level responses in the Wisconsin Card Sorting Test
S/E: Severe/extreme severity
SD: Standard deviation
WCST: Wisconsin Card Sorting Test
